# Exercise capacity, cardiovascular and metabolic risk of the sample of German police officers in a descriptive international comparison

**DOI:** 10.7150/ijms.60696

**Published:** 2021-05-27

**Authors:** Markus Strauss, Peter Foshag, Ulrich Jehn, Richard Vollenberg, Anna Brzęk, Roman Leischik

**Affiliations:** 1 Department of Cardiology I- Coronary and Peripheral Vascular Disease, Heart Failure Medicine, University Hospital Muenster, Cardiol, 48149 Muenster, Germany.; 2 Department of Cardiology, Faculty of Health, School of Medicine, University Witten/Herdecke, 58095, Hagen, Germany.; 3 Department of Medicine D, Division of General Internal Medicine, Nephrology and Rheumatology, University Hospital of Muenster, 48149 Muenster, Germany.; 4 Department of Medicine B, Gastroenterology and Hepatology, University Hospital Münster, 48149, Muenster, Germany.; 5 Department of Physiotherapy, Chair of Physiotherapy, School of Health Sciences, Medical University of Silesia, Katowice, Poland.

**Keywords:** police officers, physical fitness, risk factor, cardiovascular risk, metabolic risk, cardiorespiratory fitness

## Abstract

**Background:** The police force has the mandate to protect citizens and enforce the law for public safety. Employment in the police force is recognized as a dangerous occupation and characterized by job-related physical hazards. Therefore, good health and adequate physical condition are necessary. This study aimed to determine cardiovascular, cardiorespiratory, and metabolic risk parameters of German police officers (POs) in comparison to POs from other nations.

**Methods**: 55 male police officers from Germany participated in the survey. We examined anthropometric measurements, cardiovascular/metabolic risk factors and blood parameters. Additionally, we calculated 10-year cardiovascular risk using the Framingham Risk Score. The diagnosis of metabolic syndrome bases on the criteria of the International Diabetes Federation. We assessed cardiorespiratory status by exercise spirometry.

**Results:** The analyzed group of POs demonstrated a high prevalence of pre obesity (BMI: 28.0±3.2 kg/m², waist circumference: 97.8±12.4 cm). 61.8 % of POs showed an increased waist circumference. POs showed high prevalence of abnormal values of triglyceride (n: 24, 43,6%), and systolic (n: 29, 52,7%) and diastolic (n: 27, 49%) blood pressure. The average 10-year cardiovascular risk (by Framingham) was classified as moderate (9.6 ± 7.4 %). 32 % (n: 18) of POs in our study group were diagnosed with metabolic syndrome. Maximal relative oxygen uptake of POs was 34.1 ± 8.0 ·ml/kg^-1^ ·min^-1^.

**Conclusions:** To our knowledge, this study was one of the first to assess data on cardiovascular health, metabolic syndrome and cardiorespiratory status of police officers in Germany. The results of our study demonstrated an increased cardiovascular and metabolic risk and decreased cardiorespiratory fitness in German police officers. The present study results underline the need to implement health-promoting interventions and concepts like corporate sports activities or nutrition courses to counteract cardiovascular and metabolic risk factors. We have to reduce the subsequent development of cardiovascular and metabolic disease in this occupational group.

## Introduction

The police force has the mandate to protect citizens and enforce the law for public safety. However, working as a police officer (PO) is recognized as a dangerous occupation. The working condition includes job-related hazards as the threat of bodily injury or death, intense physical stress, and unpredictable emergencies 1. Occupational circumstances and environment are one of the most critical determinants of health 2. Socioeconomic status seems to be strongly associated with adult mortality 3. Evidence indicates that the prevalence of traditional risk factors for cardiovascular disease among the police force is high (often higher than the general population) 4. The high prevalence of typical cardiovascular risk factors, including metabolic syndrome, hypertension, hyperlipidemia, cigarette smoking, and sedentary lifestyle, in police officers is known 5. POs in the USA have a high risk of sudden cardiac arrest on duties 6. This may be attributed to working conditions of job-related stress, inadequate sleep, and unhealthy lifestyle 7.

Studies about physical activity and fitness in German POs are rare. POs are at especially high risk and have more risk factors than other occupational groups. Therefore, this study aimed to examine cardiovascular, cardiorespiratory, and metabolic parameters in German POs. A review of the literature was performed to compare our findings concerning other international published data about POs. We aimed to evaluate discrepancies in cardiovascular and metabolic risk amongst different countries.

## Methods

### Participants

Male police officers from Germany, North Rhine-Westphalia, were invited to participate in this study via internet advertisements, social media, and local corporate distribution after they responded to an official request. The recruitment of study participants was advertised at workplaces. Participation was voluntary. Inclusion criteria were working in police patrol service by shift work at the federal police force of Germany. We included a group of 55 male participants in this study. The group consisted of male Caucasians with an age range between 18 and 55 years.

Examinations were performed at baseline and at one time at the Sports Medicine Centre in Hagen (Research Sector Prevention, Public Health, and Sports Medicine, University Witten/Herdecke) by a trained clinician.

The study was carried out following the declaration of Helsinki and approved by the Witten/Herdecke Ethics Committee. Written informed consent was obtained from all participants.

### Assessment tools and procedures

We used a questionnaire to collect individual sedentary time at work (hrs), use of tobacco and alcohol, and to calculate the metabolic equivalents (METS) based on Ainsworth et al. 8. Precisely, cigarette consumption (number of cigarettes per day) and alcohol consumption (alcohol consumption in several days per week) were surveyed. Dynamic sports activities such as jogging, cycling, swimming, soccer, martial arts, and anaerobic exercise are summarized under dynamic METS and were assessed for one week. One MET corresponds to 1 kcal·kg-1·h-1.

The measurement of blood pressure was performed in a supine position with calibrated standard blood pressure cuffs. For the classification of blood pressure, the ESH/ESC guidelines 2018 for management of hypertension were used (systolic blood pressure ≥ 140 mmHg Hypertension Grade I, ≥ 160 mmHg Grade II, and ≥1 80 mmHg Grade III) 9. We measured resting heart rate by a 12-channel-ECG.

Participants were asked to fast (refrain from eating for 8 hours before the examination) before measuring body weight and having blood examinations in the morning. Bodyweight and body composition were determined using the Tanita BC-418MA segmental body composition analyzer 10. For measuring body weight, body composition, and body height, participants were instructed to wear only comfortable shorts with no other clothing or shoes. Blood serum parameters that were analyzed included: Total cholesterol, high-density lipoprotein (HDL), low-density lipoprotein (LDL), triglyceride, glycated haemoglobin (HbA1c), C-reactive protein (CRP), and lipoprotein (a). Lipoprotein (a) was measured by our laboratory (Immun - Essay La Roche).

We used the Framingham Score to calculate cardiovascular risk scores based on observations and data from the Framingham Heart Study 11. Specifically, in our investigations, we applied the 10-year Cardiovascular Disease Risk Score Calculator based on the Framingham Study 12. The following parameters were measured to calculate cardiovascular risk: age, diabetes, smoking, treated and untreated systolic blood pressure, total cholesterol, HDL levels, and BMI.

The classification used to identify individuals with metabolic syndrome is based on the International Diabetes Federation (IDF) criteria in 2005 13. The definition focuses on four entities: obesity, dyslipidaemia, hypertension, and insulin resistance. For diagnosis, obesity must be present, as well as two other criteria. The main criterion of the IDF definition, central obesity, can be assessed by waist circumference or by BMI. Waist circumference was measured at the end of expiration in a standing position in the center of the lower edge of the ribs and the upper edge of the iliac crest. The reference value of waist circumference was 94 cm. BMI was determined according to the following formula: body weight/ (height in m)^2^.

We performed spiroergometry in the following manner 14-16: After successful gas and volume calibration, a stress test was conducted beginning at 50 watts and continuously increasing by 25 watts every 2 min (ramp test). The test ended when the subject could no longer maintain the predefined cadence of 80/min or if the subject was subjectively exhausted and there was no further increase in VO2max after 20 sec. The spiroergometric analyses were conducted as previously described 15, 16. The ventilator aerobic threshold (AT) was defined as the first non-linear increase in the ventilatory equivalent for oxygen without a simultaneous increase of the ventilatory equivalent for CO2. The respiratory compensation point (RCP) was defined as the simultaneous non-linear increase of both ventilatory equivalents according to previously described recommendations 15, 16.

### Statistical Analysis

For statistical analysis, Stata/IC 13.1 for Windows was used. The anthropometric parameters, clinical characteristics, physical activity, and cardiorespiratory fitness parameters were described using the number of participants, means, standard deviations, and medians. Categorical characteristics were displayed by specifying absolute and relative frequency. Comparison of the study data with other study results was completed by using means and standard deviations.

## Results

### Study Population and Basic Characteristics

This cross-sectional study included 55 male participants. The age of the participating POs ranged from 18 to 55 years. The mean age was 45.3 ± 7.8 years (Table 1). The POs had 25.2 ± 8.4 years of professional experience varying between 5 and 41 years. The corporate sports activity of all participants was 0.58 ± 0.93 hrs/week. The whole sports activity was 4.83 ± 4.29 hrs/week. Leisure time activity with dynamic physical activities was 2838 ± 2872 METs/week.

### Cardiovascular Risk

#### Cardiovascular Risk Factors

POs demonstrated a BMI of 28.0±3.2 kg/m², which is above the upper limit of 25 kg/m². These correspond to the pre-obese/overweight range. The average cigarette consumption was 2.52 cigarettes per day. In total, 18.2% of the POs (n = 10) consumed nicotine. 76.3% of the POs had normal systolic blood pressure (RR_sys_ ≤ 139 mmHg), 23.6% of POs had a raised systolic blood pressure at rest (RR_sys_ > 140 mmHg). Diastolic elevated blood pressure values (RR_dia_ ≥ 90 mmHg) were measured in 52.2 % of all participants. The highest systolic blood pressure at rest was RR_sys_ = 170 mmHg. Table 2 represents the blood pressure measurements of our study group. The mean resting heart rate value represents 68.9 ± 11.4 beats per minute.

Table 3 shows major cardiovascular risk factors. POs showed raised total cholesterol (205.9± 40.4) and triglyceride (185.1± 145.4) levels. The parameters HDL, LDL, HbA1c, and CRP were not abnormal. 61.8% of the participants showed an abnormally large waist circumference and 23.6% had an increased BMI. By adopting the criteria of the IDF, abnormal systolic blood pressure values were detected in 52.7%, whereas abnormal diastolic blood pressure values were found in 49.1% of the cases.

For the diagnosis of metabolic syndrome, cardiovascular risk factors were taken into account 13. The examined POs showed a trend towards obesity. Abnormal values of lipid profile percentages were most frequently detected in triglycerides (abnormal values in 43.6%).

#### Cardiovascular Risk Profile by Framingham

10-year risk for cardiovascular events and the heart/vascular age (years) based on the Framingham Risk score are shown in Table 4.

The mean 10-year cardiovascular risk of POs is 9.6 ± 7.4%, whereas the mean heart/vascular age is 51.1. ± 13.9 years, exceeding their actual mean age of 45.3 years by 5.8 years.

### Metabolic Syndrome

According to the criteria of the IDF metabolic syndrome was diagnosed in 18 of 55 examined POs. This corresponded to 32.7 % (Table 5).

### Cardiorespiratory Fitness

POs showed a relative oxygen uptake (rel. VO2max) of 34.1 ± 8.0 ml/kg^-1^ ·min^-1^ (Table 6). The absolute oxygen uptake (abs. VO2max) was 3.13 ± 0.62 l/min. Maximum watt per kilogram was 3.14 ± 0.68 Wmax/kg, whereas absolute maximum watt (W) amounted to 288.5 ± 48.6. Table 6 listed the spiroergometric measurements of the study group.

## Discussion

### Cardiovascular Risk

In general, working condition at the police force is reported as a risk factor for the development of cardiovascular diseases, diabetes mellitus and higher rates of mortality 1, 4, 6. Not only are German police officers found to be at a higher risk for cardiovascular disease, when in fact, this seems to be a global problem affecting law enforcement. Various factors in terms of the working conditions are likely the cause for this. Police patrols are scheduled according to shift work. Furthermore, the majority of work time involves sitting in patrol cars, writing reports, or interviewing persons, placing the officer at a higher risk for obesity.

It is known that occupational sitting time is independently associated with the presence of obesity and other cardiovascular risk factors in men 19, 20. The time of sedentary work activity seems to be related to an increased waist circumference, elevated serum triglyceride and serum CRP levels, and lower HDL cholesterol levels, and it can accelerate the emergence and progression of cardiovascular disease 20, 21. Thomas et al. 22 could provide evidence of a link between shift work and a higher level of cardiovascular risk. Workers on night duty generally show a significantly higher BMI as well as higher levels of cholesterol and triglyceride than workers on day shift 23. It is fair to assume that these factors also play an essential role in developing cardiovascular risk to the police officers in our study.

In our group of POs, the mean blood lipid levels depicting cardiovascular risk were, increased above the upper limit of the healthy reference range except for total cholesterol (205.9± 40.4) and triglyceride (185.1± 145.4) levels. The German cohort also shows higher median results when compared to the results of international studies. A comparison of cardiovascular risk factors in published studies is shown in Table 7.

Compared to Swiss police officers 24, the analyzed German cohort showed higher mean values for BMI, waist circumference, cholesterol, LDL, and triglyceride levels.

Regarding blood pressure values in the examined POs, we observed systolic hypertension in 52.7%, and diastolic hypertension in 49.1%, based on criteria for metabolic syndrome of the IDF.

The presence of hypertension is notable in our study cohort and can be observed in the entire population and other occupational groups 30, 31. It seemed the prevalence of arterial hypertension in the group of German POs was higher compared to the general German population. Estimates show that slightly over 30% of the German population present arterial hypertensive blood pressure values 32. In international comparison, our study cohort shows values of systolic and diastolic blood pressure at the same levels as police officers from the USA, India, and Switzerland 24, 26, 28. Lower values are described in a Saudi Arabia police officer cohort 29.

One of the most important cardiovascular risk factors is smoking. The prevalence of nicotine consumption in our cohort was 18.2%. An international comparison shows that the smoking status of police officers differs significantly. However, studies show a link between shift work, specifically working the night shift and higher consumption of nicotine 23.

In this study, we analyzed lipoprotein (a) levels. Lipoprotein (a) represents an essential cardiovascular risk parameter. Elevated values of this parameter are associated with an increased cardiovascular risk profile and may - in conjunction with other cardiovascular risk factors - increase the risk for vascular diseases 33, 34. Lipoprotein (a) is a genetically determined parameter and appears to play a central role in developing coronary artery diseases and thromboembolic events 35, 36. Kamstrup et al. 34 demonstrated a 35% higher 10-year risk of suffering a myocardial infarction in males with increased lipoprotein (a) levels combined with other crucial cardiovascular risk parameters. 25.5% of German police officers show increased levels of lipoprotein (a). An international comparison to police officers of other studies is not possible because of the lack of data.

To establish the 10-year risk for cardiovascular events, the Framingham risk score was used. The 10-year risk for cardiovascular events of our analyzed POs was found to be at 9.6±7.4%.

According to a 10-year-chance on cardiovascular events being < 10%, our study population could be classified in the low-risk category 37. Due to the lack of comparative studies by using the same risk score, a direct and valid comparison of the 10-year risk stratification of cardiovascular events in POs to international studies could not be carried out. To date, there is only one study of American police officers who found there to be a lower chance of cardiovascular events in 10 years (Mean±SD: 1.2±0.5) 28.

### Metabolic Syndrome

International studies confirm an increased risk for metabolic syndrome in POs 26, 38, 39. This study used the criteria of the International Diabetes Federation (IDF) to determine metabolic syndrome 18. Metabolic syndrome is characterized by the central factor of obesity. The definition of IDF is more likely used in central Europe compared to definitions of the National Cholesterol Education Program (NCEP) and World Health Organization (WHO) 40, 41. The examined POs in this study demonstrated a mean waist circumference of 97.8 ± 12.4 cm. BMI was within the overweight range with a mean of 28.0 ±3.2kg/m^2^. Examined POs from Switzerland and India displayed lower mean abdominal circumferences and BMI values compared to our German cohort 24, 25. Mean BMI values of American POs are in the same range as our German POs, but American POs had lower mean values concerning the lipid profile (Cholesterol, LDL, and Triglyercide) 27, 28. However, increased abdominal circumference and BMI do not appear to be exclusive to our investigated German cohort; they are globally detectable in police forces.

High abdominal circumferences in the POs of our study are possibly based on sedentary work character. However, the job of POs is more sedentary as we can imagine. Administrative activities are often a relevant part of the duty and observing activities performed in a car are typical sedentary. It is known that prolonged sedentary activities are associated with higher abdominal circumference and the severity of the associated metabolic risk 42.

According to the criteria of the IDF, metabolic syndrome was detected in 32.7% of subjects in our study. Looking at various studies around the world, the prevalence of metabolic syndrome in the general population varies from 23% in France to 41% in the USA 43. Compared to the regional prevalence of metabolic syndrome of the male population of North Rhine-Westphalia (Germany), the analyzed POs were found to be at an above-average risk of metabolic syndrome (males in North Rhine-Westphalia: 22% vs. POs: 32%) 44. These results correspond to the conclusion drawn by Tharkar et al 26. It was shown that Indian POs had a significantly higher risk for metabolic syndrome than the rest of the population (57.3% vs 28.2%). However, Hartley et al 45 detected a lower prevalence of metabolic syndrome at 26.7% of the analyzed cohort in the “Buffalo Cardio-Metabolic Occupational Police Stress (BCOPS)” study. The lowest prevalence of metabolic syndrome was found by Violanti et al. 46 among POs in the USA (13.3%), and one of the highest was described for POs in Poland (53.6%) 38. Table 8 shows a comparison of the prevalence of metabolic syndrome in the professional category of “Police officers” among countries.

We can assume that there are multifactorial reasons for the high prevalence of metabolic syndrome in POs. The study by Janczura et al. 49 shows that there is a positive correlation between higher “perceived stress” (Perceived Stress Scale-10) on the job and the prevalence of metabolic syndrome in Polish police officers. Garbarino et al. 47 support this result, proving that work-related stress induces metabolic syndrome. The prevalence of metabolic syndrome can arise to 24.5% in the analyzed male police officers. Furthermore, irregular work hours, specifically those at night, can trigger sleeping disorders 50, 51. It can be assumed that a high number of German police officers also suffer from a sleeping disorder. The study by McCanlies has shown that POs who claimed to sleep less than 6 hours per 24 hours had an increased prevalence of metabolic syndrome by 150% in comparison to those POs who slept more than 6 hours per 24 hours 48.

It may seem surprising that German POs show a similarly high risk of metabolic syndrome in comparison to office workers 31, 52. This could be because both occupations conduct activities mostly while sitting. It is well known that sedentary activities carry a higher risk of metabolic syndrome 53 and cardiovascular disease 54.

Overall differences in individual status and private and professional activities exist, and there will also be differences concerning the way of life between the countries. Therefore, in the future, there is a need to perform prospective studies concerning these factors.

### Cardiorespiratory Fitness

High cardiorespiratory fitness is an important factor in the prevention and treatment of cardiovascular risk, diseases, and mortality 55, 56. In detail, higher cardiorespiratory fitness is associated with lower BMI, a lower risk for developing type 2 diabetes, and being active 57, 58. Physical fitness is an important basic requirement for police officers on duty. As we know, there are only a few studies about cardiorespiratory fitness among police officers. A direct comparison of the data investigated is challenging because of diverging parameters in age and BMI between the study groups. In our study, the examined POs reached a mean oxygen uptake of (rel. VO2max) 34.1 ± 8.0 ml/kg^-1^ min^-1^. Compared to police officers of other countries, the rel. VO2max values of German POs are the lowest. However, the comparison suffers from a lack of available study data. In comparison to international literature, German police officers show the lowest relative oxygen uptakes. Canadian police officers have the highest relative oxygen uptake (44.1±6.6 ml/kg^-1^ ·min^-1^) 59 and police officers from America and Finland also showed higher values 60, 61. A comparative presentation of relative oxygen uptake is shown in Table 9. In general, the VO2max values estimated in German police officers seemed to be lower than the range of values in healthy participants (rel. VO2max: 40.5±5.5 vs. 34.1±8.0 ml/kg^-1^ ·min^-1^) 62. Compared to recreational athletes, VO2max assessed in our study is expectedly lower (rel. VO2max: 64.7±6.7 vs. 34.1±8.0 ml/kg^-1^ ·min^-1^) 63, but almost identical compared to a mainly sedentary working group of German office workers (rel. VO2max: 34.1±8.1 vs. 34.1±8.0 ml/kg^-1^ ·min^-1^) 31. A group of professional firefighters with high degrees of physical activity at work also showed higher values of oxygen uptake (rel. VO2max: 37.3±6.3 vs. 34.1±8.0 ml/kg^-1^ ·min^-1^) 30.

### Limitations

This study contains some points that may limit the relevance of the results. The study was conducted with a small number of participants, and they only presented a small cohort of the occupational group of police officers. Second, the examined sample of police officers was from one specific area of Germany (Ruhr area) and does not represent a random sample of German police officers. Therefore, extensive prospective studies with larger sample sizes are necessary to support or refute the results.

## Conclusion

To the best of our knowledge this study was one of the first to assess data on cardiovascular health, metabolic syndrome and cardiorespiratory status of police officers in Germany. The results of our study population demonstrated an increased cardiovascular risk and low cardiorespiratory fitness in German police officers. We detected a high prevalence of elevated cardiovascular and metabolic risk factors in our cohort, especially a high prevalence of increased diastolic and systolic blood pressure at rest and increased cholesterol and triglyceride levels. A trend towards obesity could be seen in investigated individuals and in general in the police force. An increased abdominal circumference and BMI do not appear to be exclusive to our German POs cohort; they are globally detectable in this specific profession. However, the cardiovascular risk profile of our examined German cohort seems to be in the higher range in comparison to international data, and the high prevalence of metabolic syndrome amongst POs was not exclusive to our German cohort but can be globally identified.

High cardiovascular fitness is a definite job requirement for law enforcement officer. The analyzed German POs have shown the worst cardiorespiratory fitness of rel. VO2max when compared internationally. These facts show the urgency and necessity for establishing fitness-related activities to improve cardiovascular fitness in this profession.

In conclusion, the present study results demonstrate the need to implement health-promoting interventions and concepts like corporate sports activities and nutrition courses to counteract the development of cardiovascular and metabolic diseases in this occupational group.

## Figures and Tables

**Table 1:** Basic characteristics of the study population

	Police officers			
	n	Mean	SD	Median
Age (years)	55	45.3	7.8	45.0
BMI (kg/m²)	55	28.0	3.2	27.4
BSA by Mosteller (m²)	55	2.18	0.18	2.14
Muscle mass (kg)	55	69.7	7.8	69.7
Body fat (%)	55	21.4	5.6	22.2
Sports activity (hrs/week)	55	4.83	4.29	3.00
Company sports activity (hrs/week)	55	0.58	0.93	0.00

**Table 2:** Classification of systolic and diastolic blood pressure in degrees of severity

	Systolic blood pressure						
	n	Optimum	Normal	High normal	Stage 1 hypertension	Stage 2 hypertension	Stage 3 hypertension
		**≤120 mmHg**	**120-129 mmHg**	**130-139 mmHg**	**140-159 mmHg**	**160-179mmHg**	**>180 mmHg**
Police officers, n (%)	55	7 (12.7%)	19 (34.5%)	16 (29.1%)	12 (21.8%)	1 (1.8%)	0 (0%)
**Diastolic blood pressure**		**≤80 mmHg**	**80-84 mmHg**	**85-89mmHg**	**90-99 mmHg**	**100-109mmHg**	**>110 mmHg**
Police officers, n (%)	55	6 (10.9%)	22 (40.0%)	0 (0%)	18 (32.7%)	7 (12.7%)	2 (3.6%)

**Table 3:** Representation of major cardiovascular risk factors in the study population

	Police officers					
	n	Mean	SD	Median	Normal, n (%)	Abnormal, n (%)
Age (Years)	55	45.3	7.8	45.0		
**Basic dimensions**						
BMI (kg/m²)	55	28.0	3.2	27.4	42 (76.4%)	13 (23.6%)
Abdominal waist (cm)	55	97.8	12.4	96.0	21 (38.2%)	34 (61.8%)
**Blood pressure**						
RR_sys_ (mmHg)	55	127.7	12.3	130.0	26 (47.3%)	29 (52.7%)
RR_dia_ (mmHg)	55	85.7	10.2	83.0	28 (50.9%)	27 (49.1%)
**Heart rate**						
Heart rate (beats/min.)	55	68.9	11.4	67.0		
**Drink and Tobacco**						
Tobacco use (Cigarettes/day)	55	2.52	5.98	0.00	10* (18%)*	45* (82%)*
Alcohol (days a week)	55	1.52	1.61	1.00		
**Blood values**						
Cholesterol (mg/dl)	55	205.9	40.4	198.0		
HDL (mg/dl)	55	49.4	15.3	45.0	39 (70.9%)	16 (29.1%)
LDL (mg/dl)	55	129.1	37.7	119.0		
Triglyceride (mg/dl)	55	185.1	145.4	133.0	31 (56.4%)	24 (43.6%)
HbA1c (%)	55	5.5	0.4	5.4		
CRP (mg/dl)	55	0.21	0.21	0.10		
According to the criteria of Grundy et al. 17						

*Prevalence in Tobacco use (n,%).

**Table 4:** Representation of the 10-year risk of cardiovascular events and heart/vascular age of the study population

	n	Mean	SD	Median
**10-year cardiovascular risk Framingham (%)**				
Police officers	55	9.6	7.4	7.6
**Heart/Vascular Age by Framingham (Years)**				
Police officers	55	51.1	13.9	50.0

**Table 5:** Diagnosis of metabolic syndrome (number/frequency) according to IDF criteria 18

	n	No Metabolic Syndrome	Metabolic Syndrome
Police officers	55	37 (67.3%)	18 (32.7%)

**Table 6:** Spiroergometry and physical activity results of police officers

	Police officers			
	n	Mean	SD	Median
**Ventilator aerobic threshold (AT)**				
Hr	55	110.9	15.8	110.0
rel. VO2	55	17.9	5.8	17.0
%VO2max	55	52.3	13.8	52.0
W	55	133.6	41.7	132.0
**Respiratory compensation point (RCP)**				
Hr	55	133.9	21.2	132.0
rel.VO2	55	24.4	8.6	24.0
%VO2max	55	70.5	17.1	71.0
W	55	193.2	61.9	188.0
**Maximum Loading (Max)**				
Hr	55	172.4	16.2	172.0
rel.VO_2_	55	34.1	8.0	34.0
W	55	288.5	48.6	300.0
W_max/kg_	55	3.14	0.68	3.27
Dynamic METS	55	2838	2872	1978

Abbreviations: Hr: Heart rate (min); rel. VO2: Relative maximal oxygen uptake (ml/min/kg); %VO2max: Percent of absolute maximal oxygen uptake (l/min); Dynamic METS: Sports activities such as jogging, cycling, swimming, soccer, martial arts, and anaerobic exercise (MET/per week); Wmax/kg: Watt maximal per kg body weight; W: Watt.

**Table 7:** Comparison of cardiovascular risk factors in published studies of police officers

Cardiovascular risk factors							
Source	Own results	Schilling et al. 24	Thayyil et al. 25	Tharkar et al. 26	Violanti et al. 27	Everding et al. 28	Alghamdi et al. 29
	Police officers Germany	Police officers Switzerland	Police officers India	Police officers India	Police officers USA	Police officers USA	Police officers Saudi Arabia
Study cohort	55	201	823	318	58	379	160
Age (Years)	45.3±7.8	38.6±10.1	41.3±6.8	44.3±12.1	44.0±8.7	41.5±8.6	34.4±8.3
Weight (kg)	93.6±13.2		71.3±8.4		94.5±14.2		79.0±15.2
BMI (kg/m²)	28.0±3.2	25.8±3.6	23.9±2.5	25.9±4.1	29.8±5.8	28.8±3.9	27.5±5.1
Abdominal waist (cm)	97.8±12.4	91.1±11.3	85.9±11.9	92.5±11.6			
Smokers (%)	18.2		10.6	22.6			
RR_sys_ (mmHg)	127.7± 12.3	129.0± 13.0	125.2±13.4	128.3±18.8	122.6±14.9	125.1±8.6	119.5±13.9
RR_dia_ (mmHg)	85.7±10.2	85.0±10.0	82.0±9.0	85.5±12.5	81.2±10.4	85.1±7.6	79.4±11.9
Cholesterol (mg/dl)	205.9± 40.4	192.3± 38.5	207.2± 40.2	183.6± 42.8		190.2± 32.9	187.5±32.9
HDL (mg/dl)	49.4±15.3	69.2±15.4	49.1±11.3	39.8±10.5		49.4±13.3	43.9±8.6
LDL (mg/dl)	129.1± 37.7	96.2± 30.8	129.0± 38.8	106.9±38.4		115.1±28.0	119.5±24.4
Triglyceride (mg/dl)	185.1± 145.4	150.4± 106.2	143.0±56.9	177.6±116		145.8±84.7	124.5±50.9
HbA1c (%)	5.5±0.4	5.4±0.3					
10- year risk Framingham (%)	9.6±7.4					1.2±0.5	
Lipoprotein a > 30 mg/dl (%)	25.5						

**Table 8:** Prevalence of metabolic syndrome in published studies

Prevalence of metabolic syndrome			
Source	Prevalence (%)	Country	Criteria
**Police officers**			
Own results	32.7	Germany	IDF
Tharkar et al. 26	57.3	India	IDF
Thayyil et al. 25	16.8	India	Modified NCEP/ATP III
Garbarino et al. 47	24.5	Italy	IDF and NCEP/ATP III
Hartley et al. 45	26.7	USA	Modified NCEP/ATP III
McCanlies et al. 48	22.0	USA	NCEP/ATP III
Everding et al. 28	29.0	USA	IDF
Violanti et al. 46	13.3	USA	Modified NCEP/ATP III
Janczura et al. 38	53.6	Poland	IDF
Zhang et al. 39	26.0	China	IDF

**Table 9:** Published data of relative oxygen uptake (rel. VO_2max_) by police officers and healthy participants

Source	rel. VO_2max_ (ml/kg^-1^ ·min^-1^)
**Police officers**	
Own results	34.1±8.0
Pollock et al. 60	40.7±4.5
Spitler et al. 64	42.1±8.9
Sörensen et al. 61	42.8±10.1
Rhodes et al. 59	44.1±6.6
Lentz et al. 65	42.2±5.8
**Healthy participants**	
Duque et al. 62	40.5±5.5

## Abbreviations

AT: ventilator aerobic threshold
BMI: Body Mass Index
BSA: body surface area
CRP: C-reactive protein
HbA1c%: Glycated haemoglobin
HDL: high-density lipoprotein
IDF: International Diabetes Federation
LDL: low-density lipoprotein
MET: metabolic equivalent
PO: police officer
RCP: respiratory compensation point
RR_dia_: resting diastolic blood pressure
RR_sys_: resting systolic blood pressure
rel. VO2max: relative maximal oxygen uptake
abs. VO2max: absolute maximal oxygen uptake
W: absolute maximum watt
Wmax/kg: Maximum watt per kilogram
